# Binding of cellular nucleolin with the viral core RNA G-quadruplex structure suppresses HCV replication

**DOI:** 10.1093/nar/gky1177

**Published:** 2018-11-20

**Authors:** Wen-Xiu Bian, Yan Xie, Xiao-Ning Wang, Guo-Hua Xu, Bo-Shi Fu, Shu Li, Gang Long, Xiang Zhou, Xiao-Lian Zhang

**Affiliations:** 1 State Key Laboratory of Virology and Hubei Province Key Laboratory of Allergy and Immunology, Medical Research Institute and Department of Immunology, Wuhan University School of Basic Medical Sciences, Wuhan 430071, PR China; 2 Key Laboratory of Molecular Virology and Immunology, Institute Pasteur of Shanghai, Shanghai Institutes for Biological Sciences, Chinese Academy of Sciences, Shanghai, China; 3 Wuhan Institute of Physics and Mathematics, Chinese Academy of Sciences, Wuhan 430071, Hubei, China; 4 College of Chemistry and Molecular Sciences, Wuhan University, Hubei Province, Wuhan 430072, China

## Abstract

Hepatitis C virus (HCV) infection is a major cause of human chronic liver disease and hepatocellular carcinoma. G-quadruplex (G4) is an important four-stranded secondary structure of nucleic acids. Recently, we discovered that the core gene of HCV contains a G4 RNA structure; however, the interaction between the HCV core RNA G4 and host cellular proteins, and the roles of the HCV core RNA G4 in HCV infection and pathogenesis remain elusive. Here, we identified a cellular protein, nucleolin (NCL), which bound and stabilized the HCV core RNA G4 structure. We demonstrated the direct interaction and colocalization between NCL and wild-type core RNA G4 at both *in vitro* and in cell physiological conditions of the alive virus; however no significant interaction was found between NCL and G4-modified core RNA. NCL is also associated with HCV particles. HCV infection induced NCL mRNA and protein expression, while NCL suppressed wild-type viral replication and expression, but not G4-modified virus. Silencing of NCL greatly enhanced viral RNA replication. Our findings provide new insights that NCL may act as a host factor for anti-viral innate immunity, and binding of cellular NCL with the viral core RNA G4 structure is involved in suppressing HCV replication.

## INTRODUCTION

Hepatitis C virus (HCV) is an enveloped, single-stranded, positive-sense RNA virus. HCV is a major cause of human chronic liver disease and hepatocellular carcinoma. More than 185 million people worldwide (3% of the world's population) are chronically infected with HCV and at risk of developing severe liver disease and hepatocellular carcinoma (1). Until now, there has been no vaccine available against hepatitis C (1). Current treatment with interferon/ribavirin showed no response in >50% of HCV genotype 1 patients (2), and newly developed direct-acting antivirals (DAAs), albeit with impressive sustained virological response (SVR), have been associated with serious side effects (3,4). However, the interaction between host and HCV remains unclear. Thus, the search for virus-host interaction-associated factors for understanding viral immunity and pathogenesis mechanism is required.

G-quadruplex (G4) is an important four-stranded structure of nucleic acids, consisting of a G·G·G·G tetrad (G-tetrad) core and loops and including DNA G4 and RNA G4 (5–9). The G-rich G4 was found in telomeres, oncogenes, and several human important viruses, such as Epstein-Barr virus (EBV) (10–12). The motif of G4 is usually (G*x* + L_1–*N*_)3+G_*x*+_, in which *x* is 2 or 3, *N* ≤ 7, and L is one of A, G, C, T or U bases. Physiologically relevant monovalent cations, such as K^+^, play vital roles in G4 stabilization (13). DNA G4 is mainly reported in telomeres (14), promoters (15) and untranslated regions (UTRs) (16). In eukaryotes, RNA G4 structures are enriched at telomeres and within specific protein-coding transcripts (17). According to the orientation of the main chain, G4s can be divided into parallel, anti-parallel, and hybrid parallel kinds of topological structures (18).

Discovery of the role of G4s in cells and pathogens and mapping their occurrence *in vivo* have been difficult, but make up an exciting field of modern biology. Increasingly direct evidence suggests that G4 formation can serve both beneficial and regulatory roles in cells, such as forming the capping structure of telomeres, and deleterious effects, as they can impede the progression of replicative DNA polymerases. Accumulating evidence also indicates the regulation of G4 structure formation by specific proteins or chaperones that bind or promote G4 and prevent DNA damage (19). G4s have been reported to play critical cellular regulatory roles in DNA replication, genome stability, transcription, and translation and are considered as promising drug targets for anticancer therapy (10–12). Their formation from eukaryotic telomeric DNA sequences is well established, at least *in vitro*, and has more recently been the focus of attention as novel anticancer target since their formation inhibits the telomerase complex from maintaining telomere length in cancer cells.

G4s play important roles in cellular processes, which should require G4-protein interaction. A few human proteins that interact with DNA G4s have been identified. For example, helicases, such as BLM (20), FANCJ (21), FMR1 (22,23) and EBNA1 (24), have been shown to bind and unwind G4 structures. Nucleolin is reported as a multifunctional DNA/RNA binding protein. NCL has been shown to bind to various G4s (25–27), including c-myc promoter (25), HIV LTR promoter (8) and Epstein-Barr virus EBNA1 mRNA (28). While considerable knowledge has been accumulated about the interaction between DNA G4 motifs and proteins, only very little is known about protein binding to G4 forming RNA sequences (29).

Recently, we found that the HCV core gene could fold into a G4 RNA structure at positions +267 to +285 nt in the HCV 1a H77 viral genome (9), which is located in the corresponding genome region encoding core protein D1 domain. Our previous work also showed G4 ligand PDP could stabilize the core G4, and cause premature stop in HCV RNA replication and inhibition of HCV core gene translation (9). But the interaction between the HCV G4 and host cellular proteins remains elusive. Here, we further showed that a host cellular protein, nucleolin (NCL) could bind to and stabilize viral core RNA G4, and thus exert an important inhibitory effect on HCV replication in cells. We presented evidence that HCV infection could induce NCL expression, and binding of NCL with the viral core RNA G4 was involved in suppressing HCV replication and expression. Thus, our new data suggest the existence of a new paradigm of host anti-HCV innate immunity, i.e. host cell responses to HCV infection by up-regulating NCL expression, and thus which exerts an important inhibitory effect on HCV replication in cells.

## MATERIALS AND METHODS

### Searching for the G4 motif using bioinformatics methods

Putative G4 sequences (PQSs) were searched in the whole HCV genome sequence using a Perl program developed by Tan *et al.* (30). The search pattern for PQS is (G_*x*+_L_1–*N*_)3+G_*x*+_, where *x* is 2 or 3, *N* ≤ 7, the range of ‘3+’ is from 3 to 6, and L corresponds to any base (A, G, C, T or U).

### Oligonucleotides, plasmids, and antibodies

All DNA and RNA oligonucleotides were purchased from TSINGKE, Co. (Wuhan, China). Anti-NCL antibody (ab129200, Abcam, UK), anti-HCV core 1b antibody (ab2740, Abcam, UK), monoclonal mouse β-actin antibody (T0022, Affinity Bioscience, USA), monoclonal mouse GAPDH antibody (60,004-1-Ig, Proteintech, USA), goat anti-rabbit immunoglobulin G (IgG, H+L) horseradish peroxidase (HRP, S0001, Affinity Bioscience, USA), and goat anti-mouse IgG (H+L) HRP (S0002, Affinity Bioscience, USA) were used in this study.

### Cell culture and viral infection

The human hepatoma cell lines Huh7.5.1 (9,31) and Huh7.5 (32) cells were cultured in Dulbecco's modified Eagle's medium (DMEM) (Gibco, Thermo Fisher Scientific, Waltham, MA, USA) supplemented with 10% fetal bovine serum (FBS) (NATOCOR, Cordoba, Argentina). HCV Jc1 and Jc1E2^FLAG^ were provided by Professor Long (32). G4-mutated HCV (J6/JFH1-G4 Mut) was constructed according to our previous publication, with multiple point mutations at nucleotides 267, 269, 275, 278 and 279, respectively (GAGGGACTCGGCTGG sequence was changed to GA**A**G**T**ACTCG**T**CT**AC**) (9). Infection of the JFH1 2a and J6/JFH1-G4 Mut HCV cell culture (HCVcc) was performed according to our previous publication (9,31,33). In brief, Huh7.5.1 cells in 12-well plates were infected with HCVcc (MOI = 1; 0.8 × 10^6^ copies/ml) at 37°C for 4–6 h. The supernatants were discarded, and the infected cells were washed twice with phosphate-buffered saline (PBS) and incubated in DMEM containing 10% FBS for 48 to 72 h. All cultures were grown in a humidified incubator maintained at 37°C with 5% CO_2_.

### Construction of the eukaryotic expression plasmid

The cDNA of NCL (GenBank accession no. NM_005381.2) RRM_2–4_C-terminal domain was amplified and cloned in-frame into the BamHI and EcoRI sites of the eukaryotic expression vector pcDNA3.1(–)myc-his (Invitrogen, Carlsbad, CA, USA) to generate the plasmid pcDNA3.1-NCL (also named pc-NCL). The constructs were confirmed by restriction enzyme digestion along with DNA sequencing analysis.

Short hairpin RNA (shRNA) oligonucleotides against NCL (GenBank accession no: NM_005381.2) were designed by Invitrogen BLOCK-iT™ RNAi Designer and synthesized by TSINGKE, Co. (Wuhan, China). The shRNA oligonucleotides were cloned in-frame into the HindIII and ApaI sites of the pSilencer1.0-U6 (Invitrogen, Carlsbad, CA, USA) to generate the pSilencer1.0-U6-NCL (also named p-sh-NCL). The constructs were confirmed by restriction enzyme digestion along with DNA sequencing analysis. All the DNA preparations were produced using endotoxin-free purification columns (QIAGEN, Valencia, CA, USA).

### Purification of recombinant protein NCL-R3-4C

The RRM3-RRM4-C-terminal fragment of NCL cDNA (15) was amplified and cloned in-frame into the BamHI and EcoRI sites of the prokaryotic expression vector pGEX-KG to yield plasmid pGEX-NCL-R_3–4_C, which was transformed into *Escherichia coli* BL21 (DE3). The GST-NCL-R_3–4_C fusion protein was overexpressed in *E. coli* after induction with isopropyl β-d-1-thiogalactopyranoside (IPTG, 0.1 mM, 25°C, 16 h). The GST-NCL-R_3–4_C protein was purified using glutathione Sepharose 4B (Amersham Biosciences). Purified GST-NCL-R_3–4_C protein was determined by SDS-PAGE and Western blot.

### Total cellular protein extraction

Huh7.5.1 or HCVcc-infected Huh7.5.1 cells (2 × 10^5^, respectively) were seeded in a 12-well plate in DMEM supplemented with 10% FBS and incubated overnight. Cells were transfected with pcDNA3.1(–)A vector, pc-NCL, pSilencer1.0-U6 vector or p-sh-NCL, respectively, using jetPRIME^®^ transfection (Polyplus, Illkirch, France) according to the manufacturer's protocol. After 6 h, the cells were washed with PBS and supplemented with fresh growth complete medium. At 48 h post-transfection, the cells were washed, and the pellets were resuspended in radioimmunoprecipitation assay buffer (RIPA buffer, containing 50 mM Tris–HCl pH 7.4, 150 mM NaCl, 1% Triton X-100, 1% sodium deoxycholate, 0.1% SDS) according to the manufacturer's instructions. The solution was centrifuged at 12 000 g for 20 min, and the supernatant was subsequently used for SDS-PAGE and Western blot. The concentration of the protein was measured using a BCA kit (Beyotime, Shanghai, China).

### In-gel digestion and LC–MS/MS protein identification

Gel processing and in-gel digestion were performed as described in the previous study (34). In brief, gel slices were destained, followed by the addition of 10 mM of dithiothreitol (DTT)/50 mM NH_4_HCO_3_, iodoacetamide and trypsin (0.025 μg/μl trypsin/10 mM NH_4_HCO_3_) solution to reduce, alkylate and digest, respectively. After digestion, the peptides were extracted from the gel cubes with 150 μl of extraction buffer (60% acetonitrile/5% formic acid) and incubated for 15 min at room temperature (RT) in a shaker. The supernatant was transferred to a new tube, and the gel pieces were further extracted with 100 μl of extraction buffer. Prior to LC–MS, the extracts were concentrated in a vacuum centrifuge and desalted with Millipore ZipTip C18.

Peptides were detected by high-performance liquid chromatography/electrospray ionization tandem mass spectrometry (HPLC–ESI-MS/MS), which was carried out on a hybrid quadrupole-time-of-flight (TOF) LC/MS/MS mass spectrometer (TripleTOF 5600+, AB Sciex, Canada) equipped with a nanospray source. Peptides were first loaded onto a C18 trap column (5 μm, 5 × 0.3 mm, Agilent Technologies) and then eluted into a C18 analytical column (75 μm × 150 mm, 3-μm particle size, 100 Å pore size, Eksigent). Mobile phase A (3% DMSO, 97% H_2_O, 0.1% formic acid) and mobile phase B (3% DMSO, 97% ACN, 0.1% formic acid) were used to establish a 30-min gradient, which comprised 0 min in 5% B, 15 min of 5–35% B, and 1 min of 35–80% B; the gradient was maintained in 80% B for 5 min, followed by 0.1 min of 80–5% B, and a final step in 5% B for 8.9 min. A constant flow rate was set at 300 nl/min. MS scans were conducted from 350 to 1500 amu, with a 250-ms time span. For MS/MS analysis, each scan cycle consisted of one full-scan mass spectrum (with *m/z* ranging from 350 to 1500 and charge states from 2 to 5) followed by 40 MS/MS events. The threshold count was set to 120 to activate MS/MS accumulation, and former target ion exclusion was set to 18 s. Raw data from TripleTOF 5,600+ were analyzed with ProteinPilot Software. Data were searched against the UniProt human reference proteome database using the following parameters: sample type, identification; cys alkylation, iodoacetamide, digestion, and trypsin. Search effort was set to rapid ID.

### Biotin-G4 pull-down assay

Biotin-G4 pull-down assay was performed as follows (29). First, *in vitro* synthetic biotin-HCV 1a core RNA G4 (5′-biotin-GGGCUGCGGGUGGGCGGGA-3′) (9) or G4-modified RNA (5′-biotin-GGACUGCGUGUGAGCGGGA-3′, with the underlined letters representing modifications at G269A, G275U and G279A) (10 μg), was incubated in 10 mM Tris–HCl (pH 7.0) buffer containing 100 mM KCl to form the G4 structure and then incubated with streptavidin-agarose (Thermo Fisher, 20347) (20 μl) at 4°C for 2 h. It was then washed 3 times with PBS at 4°C. Next, total cell lysates (2 mg of proteins) with phenylmethane sulfonyl fluoride (PMSF, ST506, Beyotime Biotechnology, China) were incubated with biotin-G4-streptavidin-agarose at 4°C overnight. The precipitated samples were washed 3 times with PBS as described above to remove unbound proteins and then analyzed by SDS-PAGE. The bands of pulled proteins were extracted from the SDS gel and prepared for identification with a hybrid quadrupole-TOF LC/MS/MS mass spectrometer (TripleTOF 5600+, AB Sciex, Redwood, CA, USA) equipped with a nanospray source.

### Western blot analysis

Total proteins of Huh7.5.1 or HCVcc-infected Huh7.5.1 cells (2 × 10^7^, respectively) were extracted with RIPA and then quantified using the BCA Protein Assay Kit (Beyotime, China). Next, 30 μg of each lysate was loaded onto SDS-PAGE gels. The electrophoresed proteins were transferred to PVDF membranes (Millipore, USA). The membranes were blocked with 5% non-fat milk and incubated with diluted anti-core antibody (1:1000; Abcam; UK) and anti-NCL antibody (1:10,000; Abcam; UK), followed by HRP-conjugated secondary antibodies (1:3000, Affinity bioscience; USA). β-Actin (T0022, Affinity bioscience; USA) or GAPDH (60 004-1-Ig, Proteintech; USA) was used as the endogenous control.

### Quantification of viral and NCL mRNA by RT-qPCR

Huh7.5.1 or HCVcc-infected Huh7.5.1 cells (2 × 10^5^, respectively) were transfected with 1 μg of different plasmids (pcDNA3.1(–)A, pc-NCL, pSilencer1.0-U6, p-sh-NCL). The total RNAs were extracted using TRIzol reagent (Invitrogen, Carlsbad, CA, USA). First-strand cDNA was synthesized from the total RNA using the ReverTra Ace-α First strand cDNA Synthesis Kit (Toyobo, Osaka, Japan). RT-qPCR was conducted in optical tubes in a 96-well microtitre plate (Applied Biosystems) with an ABI Step One Plus™ Real-Time PCR system (Applied Biosystems), and the fluorescence signals were generated and recorded during each PCR cycle. The relative mRNA expression of HCV RNA levels at 5′ UTR and NCL mRNA were quantitated using RT-qPCR with the SYBR Green real-time PCR Master Mix kit (Toyobo, Osaka, Japan). The fold change was calculated as 2^−dCt^, where dCt = Ct (experimental group) – Ct (control group). The primers for HCV RNA at 5′ UTR were HCV-F: 5′-RAYCACTCCCCTGTGAGGAAC-3′ and HCV-R: 5′-TGRTGCACGGTCTACGAGACCTC -3′ (R represents A or G; Y represents C or G) (9,35). The primers for NCL were NCL-F: 5′-GAAGCCAGCCATCCAA-3′ and NCL-R: 5′-TCTGCCACCAAATCCT-3′. GAPDH ((glyceraldehyde 3-phosphate dehydrogenase), GAPDH-F: 5′-GAAGGTGAAGGTCGGA GTC-3′ and GAPDH-R: 5′GAAGATGGTG ATGGGATTTC-3′) was used as an endogenous control.

### Confocal immunofluorescence

To analyze the colocalization between synthetic G4 or G4-Mut RNA and NCL, G4-specific antibody BG4 was prepared according to a previously described protocol (14). Huh7.5.1 cells were plated in confocal dishes (NEST Biological Technology Co., Ltd., Shanghai, China) at a density of 1 × 10^6^ cells/dish, and after 24 h, Huh7.5.1 cells were transfected with 100 pmol FAM-labelled G4 or G4-Mut oligo RNA using jetPRIME^®^ transfection (Polyplus, Illkirch, France). 24 h later, Huh7.5.1 cells were stimulated with or without PDP/PDS/TMPyP4 (with 2.5 μM for each), then incubated at 37°C for 4 h. Later, cells in confocal dishes were washed 3 times with PBS. Subsequently, the cells were incubated with BG4-Flag (4 μg/ml) at 4°C overnight, followed by the addition of mouse anti-Flag (1:500 dilution, M185-3L, MEDICAL&BIOLOGICAL LABORATORIES CO., LTD) and rabbit anti-NCL antibody (1:100 dilution, ab129200, Abcam) at RT for 1 h. The cells were washed 3 times with PBS, followed by addition of anti-mouse rhodamine-conjugated IgG (1:50 dilution, SA00007-1, Proteintech) and anti-rabbit Alexa Fluor 647-conjugated IgG (1:200 dilution, ab150079, Abcam). After incubation at RT for 1 h and washing with PBS, nuclei were stained with DAPI (1:1000, Beyotime, China) in 1× PBS for 5 min at RT. The excitation laser used for the FAM group was 488 nm. Rhodamine was excited with a 568 nm diode laser and detected using the 590 nm long pass filter (LP). Alexa Fluor 647 was excited at 647 nm and detected with a 647 nm LP.

To analyze the colocalization between G4 RNA from HCV whole genome and NCL, XbaI restricted digestion of the plasmid pJ6/JFH1 at the 3′ end of the HCV cDNA was performed, and the linearized plasmid was *in vitro* transcribed to generate full-length HCV genomic RNA. Moreover, pJ6/JFH1-G4 Mut plasmid containing a G4-mutated sequence in the C gene was prepared using overlapping extension PCR (OE-PCR) (9). The transcribed J6/JFH1 or J6/JFH1-G4 Mut RNA were transfected into Huh7.5.1 cells using Oligofectamine reagent (Invitrogen), respectively, according to the manufacturer's instructions, followed by fixation, permeabilization and blocking according to a previous protocol (14). Then, Huh7.5.1 cells were incubated with FAM-labelled ASO (antisense oligonucleotides) complementary to a sequence upstream of the target G4 site. Cells in confocal dishes were washed three times with PBS, Subsequently, the cells were incubated with BG4-flag, followed by the addition of mouse anti-Flag, and then same treatment as above described.

Next we used a specific aptamer ZE18 ‘antibody’ against HCV E2 to detect the effect of NCL on the HCV expression in Huh7.5.1 cells infected with alive viral particles (36). Huh7.5.1 cells were seeded and grown to 70–90% confluence at the time of transfection in confocal dishes (NEST Biological Technology Co., Ltd., Shanghai, China) and transfected with different plasmids using jetPRIME^®^ transfection (Polyplus, Illkirch, France) according the manufacturer's instructions. After 6 h of transfection, the cells were replenished with fresh medium and infected with HCV for 48 h. Next, the FAM-ZE18 aptamer ‘antibody’ of HCV was added to the medium and incubated in a 37°C incubator for 30 min in the dark. Cells in confocal dishes were washed 3 times with PBS, followed by fixation, permeabilization and blocking using a previous protocol (9). DAPI was added for 5 min at RT. Cells were washed with PBS and detected using a Leica-LCS-SP8-STED.

### Circular dichroism (CD) and CD melting studies

The 6 different putative G4 sequences were synthesized, HCV1a core/E1/NS3/NS4B/NS5A/NS5B RNA G4s, and each G4s (8.0 μM) was dissolved in 10 mM of Tris-HCl buffer (pH 7.0) containing 100 mM of K^+^. CD experiments were performed on an Applied Photophysics Chirascan (Applied Photophysics Ltd., Leatherhead, Surrey, UK) equipped with a Peltier temperature controller. For CD-melting measurement, the ellipticity at 264 nm was monitored on continuous heating at 1°C/min between 10 and 95°C. And the melting point (*T*_m_) corresponding to the mid-transition temperature was calculated by a Sigmoid Curve in the software of the equipment itself. The function used was as follow: Ellipticity = Ab + ((At – Ab)/(1 + exp((*x*_0_ - *x*)/*w*))) and the *x*_0_ represents the *T*_m_.

### Fluorescence resonance energy transfer (FRET) kinetic assay

Dual-labeled G4 RNA probe (HCV core RNA G4-dual) containing a donor fluorophore (FAM) and an acceptor fluorophore (TAMRA) (at a final concentration of 200 nM) were prepared in 10 mM Tris-HCl buffer (pH 7.0) with 100 mM KCl and equilibrated at 25°C for 30 min. For the FRET kinetic assays, the ASO sample (AS-HCV core RNA G4 at a final concentration of 2.0 mM) was mixed with the above probe samples and different amounts of NCL-R_3–4_C (0–8.0 molar equiv.) at *t* = 0. Fluorescence detection was conducted at 25°C in kinetics mode. The F-4600 spectrometer was used with a 1-cm path length cell. The excitation and emission wavelengths were set to 494 and 590 nm, respectively.

### NMR (nuclear magnetic resonance) spectroscopy

^1^H NMR spectra was recorded at 298 K using a 700-MHz Bruker Avance III HD spectrometer equipped with a triple resonance 5 mm HCN-cryoprobe. Water suppression was achieved using the excitation sculpting method. The RNA samples (HCV core RNA G4: 5′-GGGCUGCGGGUGGGCGGGA, at a final concentration of 0.5 mM, and ASO-HCV core RNA G4: 5′-UCCCGCCCACCCGCAGCCC, at a final concentration of ASO was 0.1 mM) were dissolved in 10 mM PBS (pH 7.0) containing 100 mM KCl and 10% D_2_O. The RNA samples were annealed after heating at 90°C for 4.0 min and slowly cooled to 25°C.

### Gradient purification of HCV particles and affinity purification using FLAG-specific antibodies

A concentrated culture supernatant from Jc1 or Jc1E2^FLAG^ viruses was prepared according to previous report (32) and collected HCV particles were used for TCID_50_ (TCID50, 50% tissue culture infective dose) analysis.

Concentrated virus HCV Jc1 or Jc1E2^FLAG^ (all derived from the J6/JFH1 chimera Jc1 [genotype 2a/2a]) obtained from transfected Huh7.5 cells were purified with a 50 μl bed volume of a FLAG M2 affinity gel (Sigma) on a spinning wheel for at RT for 3 h (32).

### Quantification of HCV infectivity

Infectivity titers (TCID_50_) were determined by a limiting dilution assay as previously described (37). In brief, Huh7.5 cells were seeded into 96-well plates and fixed 3–4 days after infection. For immunohistochemistry, we used an antibody specific for the JFH1 NS3 helicase (2E3, provided by H Tang, Florida State University) at a dilution of 1:100. Bound antibody was detected with a peroxidase-conjugated secondary antibody specific to murine IgG (Sigma) diluted 1:200 in PBS. Virus infectivity was determined by counting the number of HCV-positive foci in each well.

### Statistical analysis

Data are presented as the mean ± SEM and analyzed using GraphPad Prism V.5.00 software (GraphPad Software, San Diego, CA, USA). Differences between groups were tested by *t*-test or one-way ANOVA followed by the Newman-Keuls post hoc test. Two-sided *P*-values less than 0.05 were considered statistically significant (*P < 0.05, **P < 0.01, ***P < 0.001).

## RESULTS

### HCV core G4 preferentially has a stable parallel G4 structure

**Figure 1.**
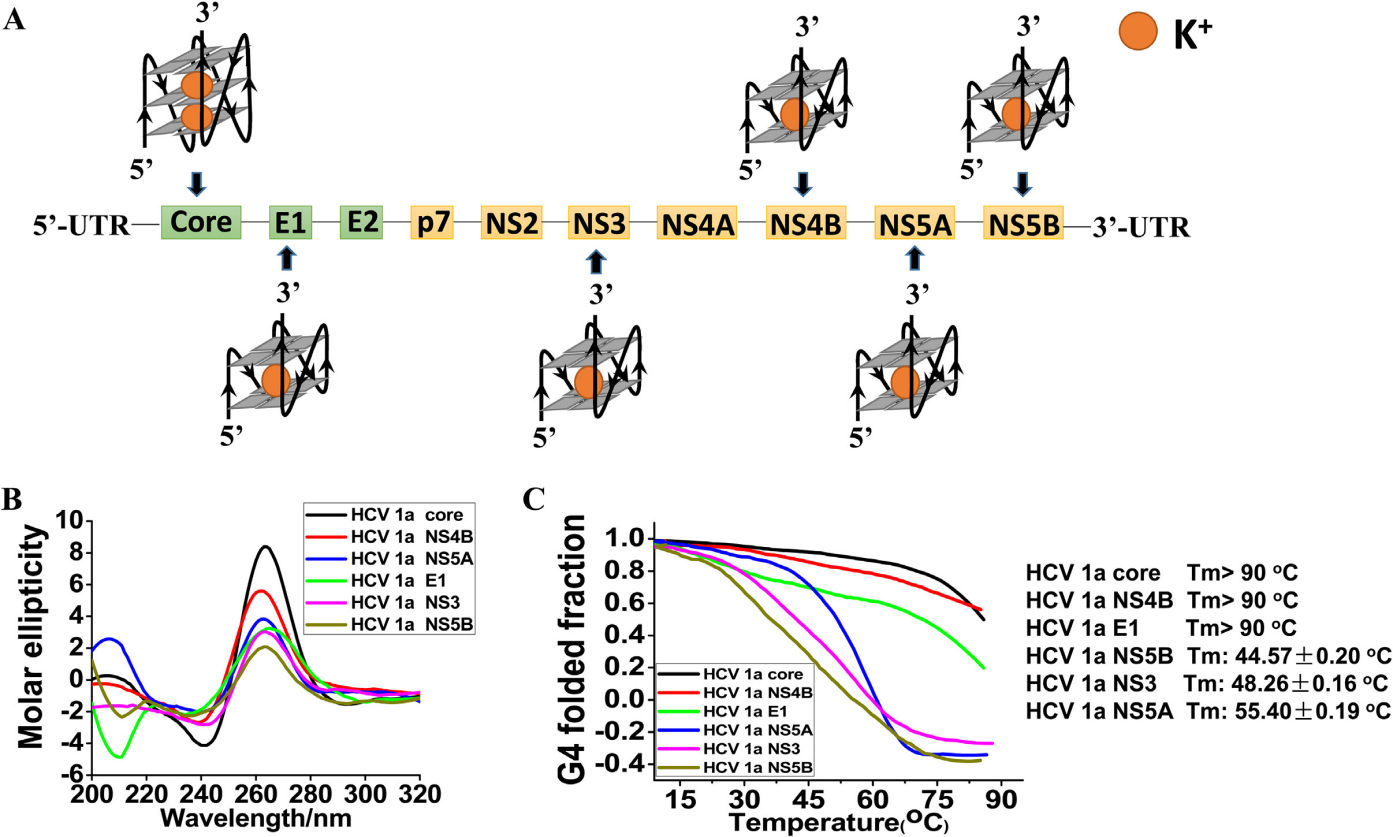
Six representative conserved PQS sites, CD and Tm melting curves. (**A**) Six conserved PQS (core/E1/NS3/NS4B/NS5A/NS5B) sites of the HCV 1a H77 genome. (**B**) Synthetic RNA PQS samples (8.0 μM) annealed in 10 mM Tris–HCl buffer (pH 7.0) containing 100 mM KCl were heated to 90°C and cooled to RT at a rate of 1°C min^–1^. The CD spectra were recorded at 25°C with a scan range from 320 to 200 nm. (**C**) The *T*_m_ thermal stability of each G4 RNAs was measured by recording the molar ellipticity at 264 nm as a function of temperature. Tm values for each oligonucleotide were calculated as described in Materials and Methods.

We first found five putative G4 sequence (PQS) motifs in E1, NS3, NS4B, NS5A and NS5B of the HCV 1a H77 positive strand RNA viral genome using bioinformatics methods as described in Materials and Methods (Supplementary Table S1 and Figure 1A), and compared them with HCV core G4 (Figure 1B). All six PQSs showed a positive peak at 267 nm and a negative peak at 238 nm, which is indicative of the presence of a parallel G-quadruplex structure based on the CD spectra (Figure 1B). The Tm values for each PQS were found to be 44.57 ± 0.20°C for NS5B, 48.26 ± 0.16°C for NS3, 55.40 ± 0.19°C for NS5A, and >90°C for core, NS4B and E1 (Figure 1C). The core G4 had one of the highest Tm values (Figure 1C), which suggests that core G4 was preferentially stable among these potential G4s in the HCV genome (Figure 1C). Additionally, HCV core G4 has a typical parallel G4 structure with 3 stacked planar G-quartets and short-length intervening loop sequences, and HCV core RNA1a (5′-GGGCUGCGGGUGGGCGGGA-3′), RNA1b (5′-GGGCAUGGGGUGGGCAGGA-3′) and RNA2a (5′-GGGAAUGAGGGACUCGGCUGGGCAGGAUGG-3′) were among the most frequent sequences observed to display a high potential of G4 formation (9).

### The HCV core RNA G4 specifically binds to cellular NCL

**Figure 2.**
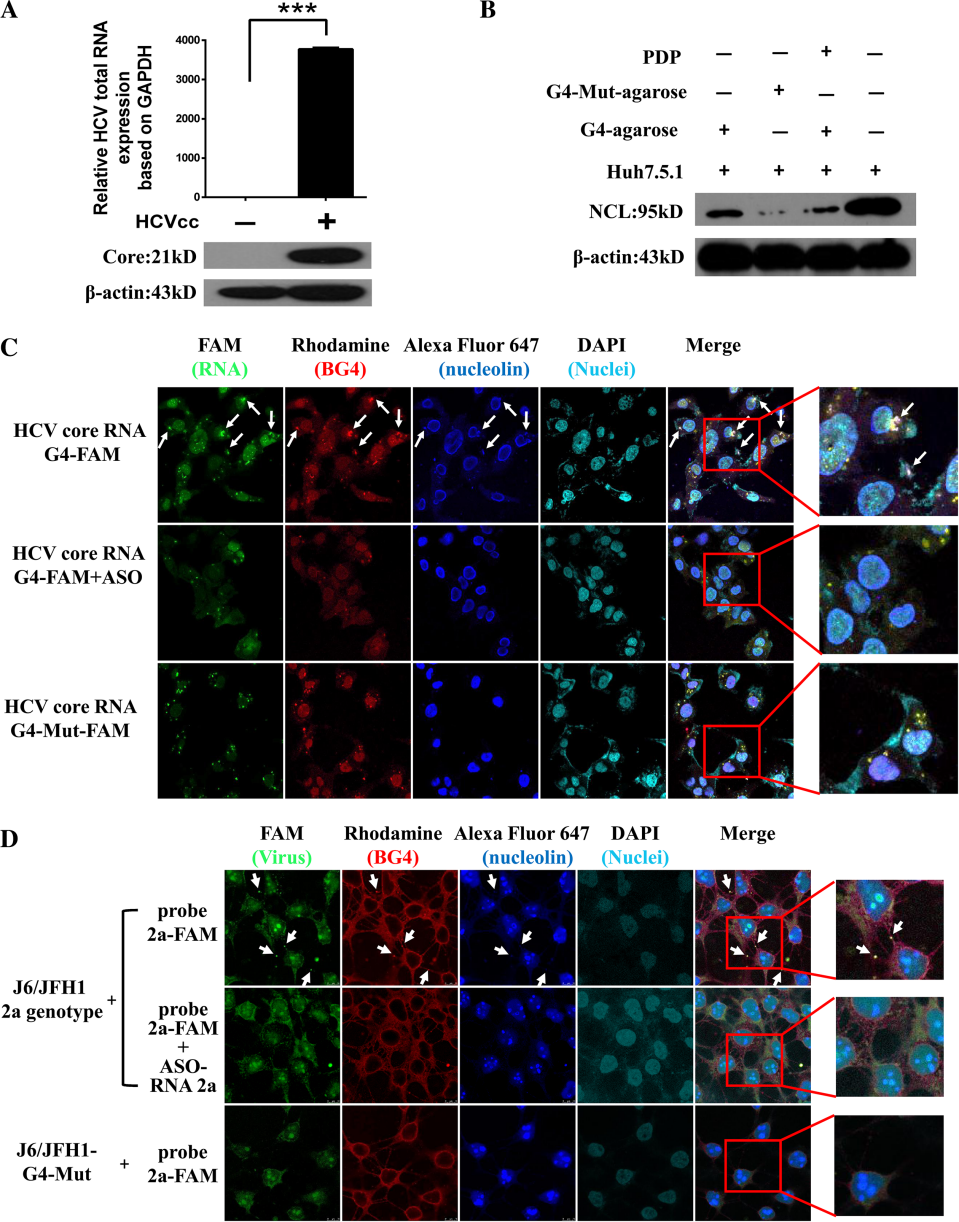
Direct interaction and colocalization between HCV core RNA G4 and NCL in Huh7.5.1 cells. (**A**) RT-qPCR and Western blot analysis of HCV RNA and core protein expression. (**B**) G4 pull-down and Western blot. The fourth lane represents cell lysates directly used for western blot. β-actin was used as an internal control. (**C**) Colocalization of HCV core RNA G4 with NCL in Huh7.5.1 cells by confocal immunofluorescence. (**D**) Analysis of colocalization of wild-type HCV and modified G4 HCV with NCL in Huh7.5.1 cells by confocal immunofluorescence. Images in C and D with magnification 63×.

Next, we identified any cellular or viral protein ligands that bound to core RNA G4. Currently, the most commonly used infectious HCV culture system is based on JFH1 (Japanese fulminant hepatitis 1, genotype 2a) (38), which undergoes efficient replication in Huh-7 cells and other cell lines (39). Using the JFH1-infected Huh7.5.1 cell model, reverse transcription real-time quantitative polymerase chain reaction (RT-qPCR) was performed, and HCV RNA levels were determined relative to the transcription of GAPDH in host cells (Figure 2A, upper). Western blot analysis was performed to determine the core protein levels of JFH1-infected Huh-7.5.1 cells using the commercial anti-HCV core antibody (Figure 2A, Lower). We further performed a biotin-conjugated synthetic-G4 plus streptavidin–agarose pull-down assay (29) with Huh7.5.1/JFH1-Huh7.5.1 cell lysates, and then subjected this to liquid chromatography–mass spectrometry (LC–MS/MS) analysis. We identified the NCL protein, which had the highest protein score according to LC-MS/MS analysis, as the most probable host binding partner for core G4 (Supplementary Table S2). Further validation with pull-down and Western blot assay confirmed the binding of NCL to G4 (Figure 2B). However, biotin-conjugated synthetic modified-G4 (G4-Mut) could not pull down NCL (Figure 2B). These results suggested that the binding of NCL to core G4 depended on the specific G4 structure. PDP is a small compound that can bind to and stabilize HCV core G4 (9). In the competition assay, the binding of core RNA G4 to NCL was decreased by a PDP competitor (Figure 2B). This finding suggests that PDP and NCL competitively bind G4. The report by Lista *et al.* has also described a competition between a G4 ligand and NCL for the binding to G4 of Epstein-Barr virus EBNA1 mRNA (28).

Because G4 structures have been visualized in human cells using antibody-based fluorescent imaging (14,40,41), we further applied G4 antibody-based fluorescence imaging strategy (14,40,41) to confirm that the core G4 motif was targeted by NCL in living Huh7.5.1 cells (Figure 2C). A well-developed G4 binding antibody BG4 (14) was prepared and used (Supplementary Figure S1). We first transfected FAM-labelled target RNAs into Huh-7.5.1 cells. After fixation and permeabilization as illustrated in Materials and Methods, amplified red fluorescence indicating G4 formation (the 2nd column of images), and blue color indicating NCL (the 3rd column of images) were generated, according to a previously described method (40). DAPI staining represents nuclei. As shown in the merged channel (Figure 2C), overlapping regions of red, green and blue generated white speckles (white arrows) in the cytoplasm of cells, indicating colocalization between core RNA G4 and NCL in Huh7.5.1 cells. However we observed that the colocalization was decreased using antisense oligonucleotides (ASO) (targeting the G4 site) treatment and a G4-modified sequence (G4-mut RNA-modified-FAM) (Figure 2C).

To further verify whether the full HCV genome forms G4 at the target site in cells, we transfected HCV (genotype 2a J6/JFH1, or G4-modified virus, named as G4-Mut) genome RNA into Huh-7.5.1 cells as illustrated in Materials and Methods and confirmed by colocalization between wild-type HCV (green FAM-aptamer ZE18 against HCV E2, the 1st column of the images), G4 (red BG4 probe, the second column of images) and NCL (the 3rd column of images) (Figure 2D). We observed that the colocalization was decreased using ASO treatment or G4-modified virus (Figure 2D). These findings provided strong evidence that NCL directly interacted with HCV core RNA G4 under physiological conditions.

**Figure 3.**
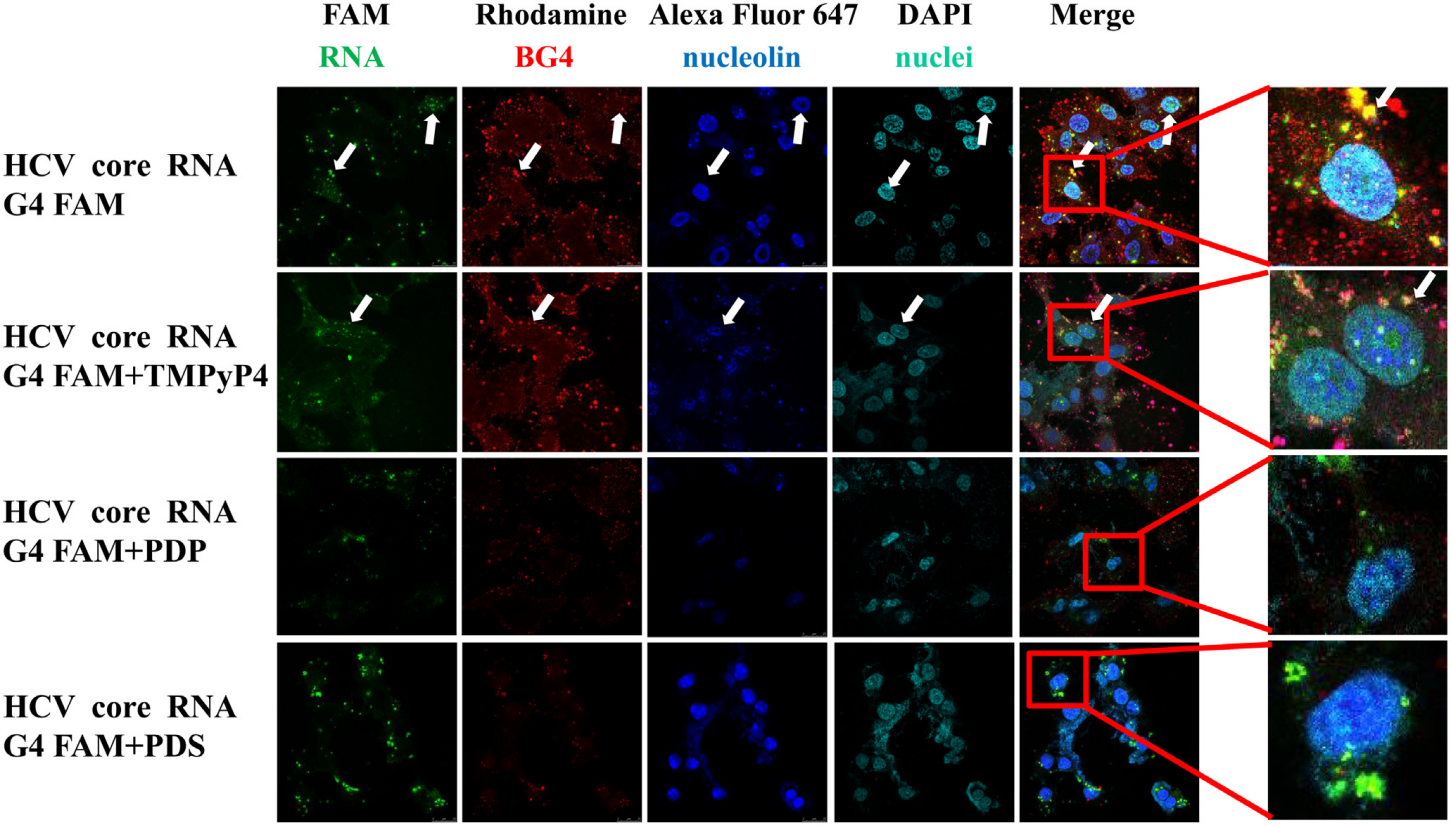
The effects of G4 ligands on the colocalization of NCL and HCV core RNA G4 in Huh7.5.1 cells by confocal immunofluorescence analysis. Confocal immunofluorescence images with magnification 63×.

In order to detect whether G4 ligands could prevent the binding of NCL to the HCV core RNA G4s, we assessed the competition between NCL and G4 ligands (PDP, TMPyP4 and PDS) for binding HCV core RNA G4. Our results showed that colocalization between HCV core RNA G4 and NCL in Huh7.5.1 cells was significantly decreased in the presence of PDP and PDS (Figure 3), since no/few white speckles (merged regions of red, green and blue generated white speckles, indicated as white arrows) were observed in the PDP and PDS groups (Figure 3). However TMPyP4 group still has white speckles (white arrows) (Figure 3), suggesting TMPyP4 has limited/no competition effect. These results suggest that different G4 ligands might have different effects on the binding of NCL to HCV core G4. PDP and PDS have similar effects for preventing the binding of NCL to the HCV core RNA G4s, while another G4 ligand TMPyP4 has no effect for it.

### NCL-R_3-4_C domain can stabilize core RNA G4 and inhibit the trap by the corresponding ASO

**Figure 4.**
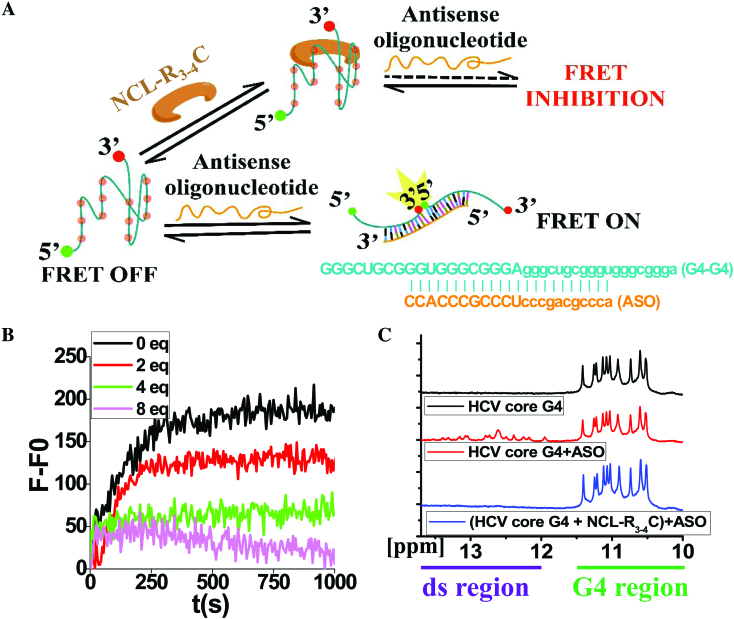
NCL-R_3–4_C can stabilize target core RNA G4 and inhibit the trap by the corresponding ASO. (**A**) Schematic depiction of the inhibition of FRET. (**B**) Time course of the fluorescence intensity (*F* – *F*_0_) of a dual-labelled probe with increasing amounts of NCL-R_3–4_C. The final concentrations of HCV core RNA G4-dual and AS-HCV core RNA G4 were 200 nM and 2.0 mM, respectively. *F* – *F*_0_ = (real time fluorescence intensity – initial fluorescence intensity)/initial fluorescence intensity. (**C**) G4 structure of HCV core RNA G4 evidenced by ^1^H NMR. The final concentrations of HCV core RNA G4 and ASO were 0.5 and 0.1 mM, respectively.

Analysis of the amino acid sequence of NCL has revealed the presence of three different structural domains (Supplementary Figure S2) (42). The central domain contains four RNA recognition motifs (RRMs). C-terminal GAR stands for glycine/arginine-rich domain. RRM3-4 and GAR domain had potential for binding to G4 (15). We demonstrated that the addition of purified NCL-R_3–4_C protein (Supplementary Figure S3) increased the G4 stability and therefore inhibited the trap by the corresponding ASO (Supplementary Table S3) using a fluorescence resonance energy transfer (FRET) kinetic assay (Figure 4A, B) and NMR (Figure 4C). The FRET assay showed that as the amount of NCL-R_3–4_C protein increased, the fluorescence intensity (*F* – *F*_0_) gradually decreased (Figure 4B). The imino proton peaks were within the 10.0–11.5 ppm region that was highly characteristic of Hoogsteen hydrogen bonds of G-quartets by ^1^H NMR analysis (43). Our results showed that the HCV core RNA G4 had well-resolved imino peaks within the 10.0–11.5 ppm region (Figure 4C), indicating the formation of G4 structures. Additional peaks appeared in the lower field of 11.5–13.5 ppm (red spectrum, Figure 4C) in the presence of ASO, suggesting the partial conversion of G4 into double-stranded RNAs. These data suggested that binding of ASO to G4 disrupted the G4 structure (double-stranded regions appeared, G4 region reduced). However, when HCV core RNA G4 was pre-incubated with NCL-R_3–4_C protein and followed by the addition of ASO, the double-stranded region peaks between 11.5 and 13.5 ppm were not observed (blue spectrum) compared with the red spectrum. These experiments indicated that NCL-R_3–4_C could bind to and stabilize the HCV core RNA G4, and further addition of ASO could not disrupt the G4 structure to form double-stranded RNAs (Figure 4C).

### NCL is associated with HCV particles

**Figure 5.**
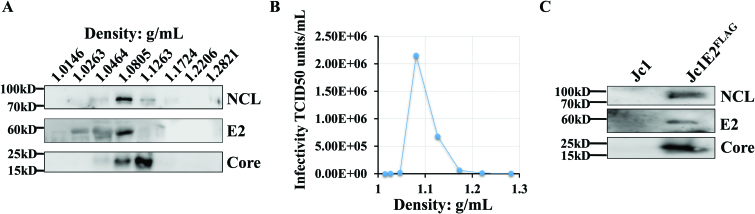
NCL is associated with HCV particles. Density gradient centrifugation analysis (**A**) and TCID50 (**B**). (**C**) Each purified HCV virion was assessed by Western blot. Jc1 virus was used as a parallel control with no flag tag.

Because we found that NCL interacted with core RNA G4, we hope to identify whether NCL is involved in HCV particles. A concentrated HCV Jc1E2^FLAG^ virus stock prepared as previously reported (32) were subjected to density gradient centrifugation, and western blot. We observed NCL and viral Core/E2 protein bands at same density of 1.0805 g/ml (Figure 5A), suggesting that NCL may be involved with HCV particles. The infectivity of HCV was further determined by calculating the 50% tissue culture infective dose (TCID50). TCID50 was also enriched and confirmed at a density of 1.085 g/mL for Jc1E2^FLAG^ (Figure 5B), which was equivalent to the fractions of NCL and core/E2 shown in Figure 5A. In the purified Jc1E2^FLAG^ preparations, core, E2, and NCL were readily detected in the viral particles by western blot (Figure 5C). These data suggest that NCL is associated with core and present in the purified HCV virion.

### NCL suppresses HCV replication and expression in Huh7.5.1 cells

**Figure 6.**
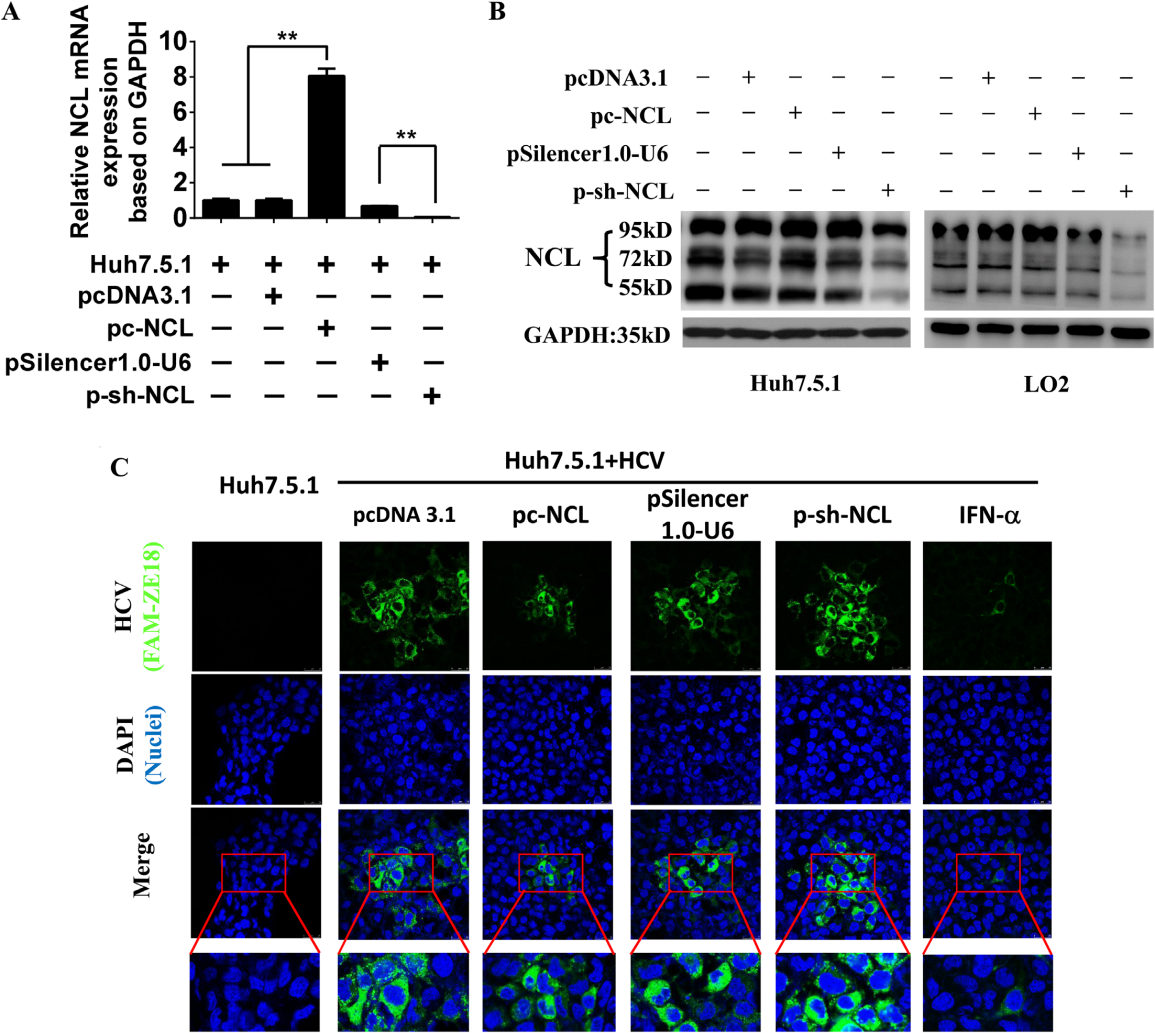
NCL suppresses HCV expression. RT-qPCR and western blot analysis of NCL expression after NCL overexpression or silencing. RT-qPCR (**A**) and western blot (**B**) analysis of NCL mRNA and protein expression after transfection of pc-NCL or p-sh-NCL in Huh7.5.1 cells or LO2 cells. pc-NCL vector group versus Huh7.5.1 cells or pcDNA3.1 vector group, **P < 0.01. p-sh-NCL vector group versus pSilencer1.0-U6 vector group, ***P < 0.001. (**C**) NCL suppresses HCV expression by confocal immunofluorescence analysis. Interferon-α (IFN-α, 150 ng/ml), a well-known effective cytokine for HCV treatment, was used as a positive control.

We further assessed the effect of NCL on HCV expression in Huh7.5.1 cells with confocal immunofluorescence assay. We analyzed the NCL mRNA and protein expression after transfection of pc-NCL or p-sh-NCL in Huh7.5.1 cells or LO2 human normal liver cells by RT-qPCR (Figure 6A) and Western blot (Figure 6B) analysis. Our results showed that NCL was successfully overexpressed or silenced after transfection of pc-NCL or p-sh-NCL, respectively. We used a specific aptamer ZE18 ‘antibody’ against HCV E2 to detect the effect of NCL on the HCV expression in Huh7.5.1 cell. We observed less green colour in HCV in the overexpression of NCL (pc-NCL group) compared to the empty vector pcDNA3.1 group, and more green colour in HCV when silencing NCL (p-sh-NCL group) compared to the pSilencer1.0-U6 empty vector group (Figure 6C). Interferon-α (IFN-α) was used as an anti-viral drug positive control (Figure 6C). These data indicate that overexpression of NCL suppresses HCV expression in Huh7.5.1 cells.

**Figure 7.**
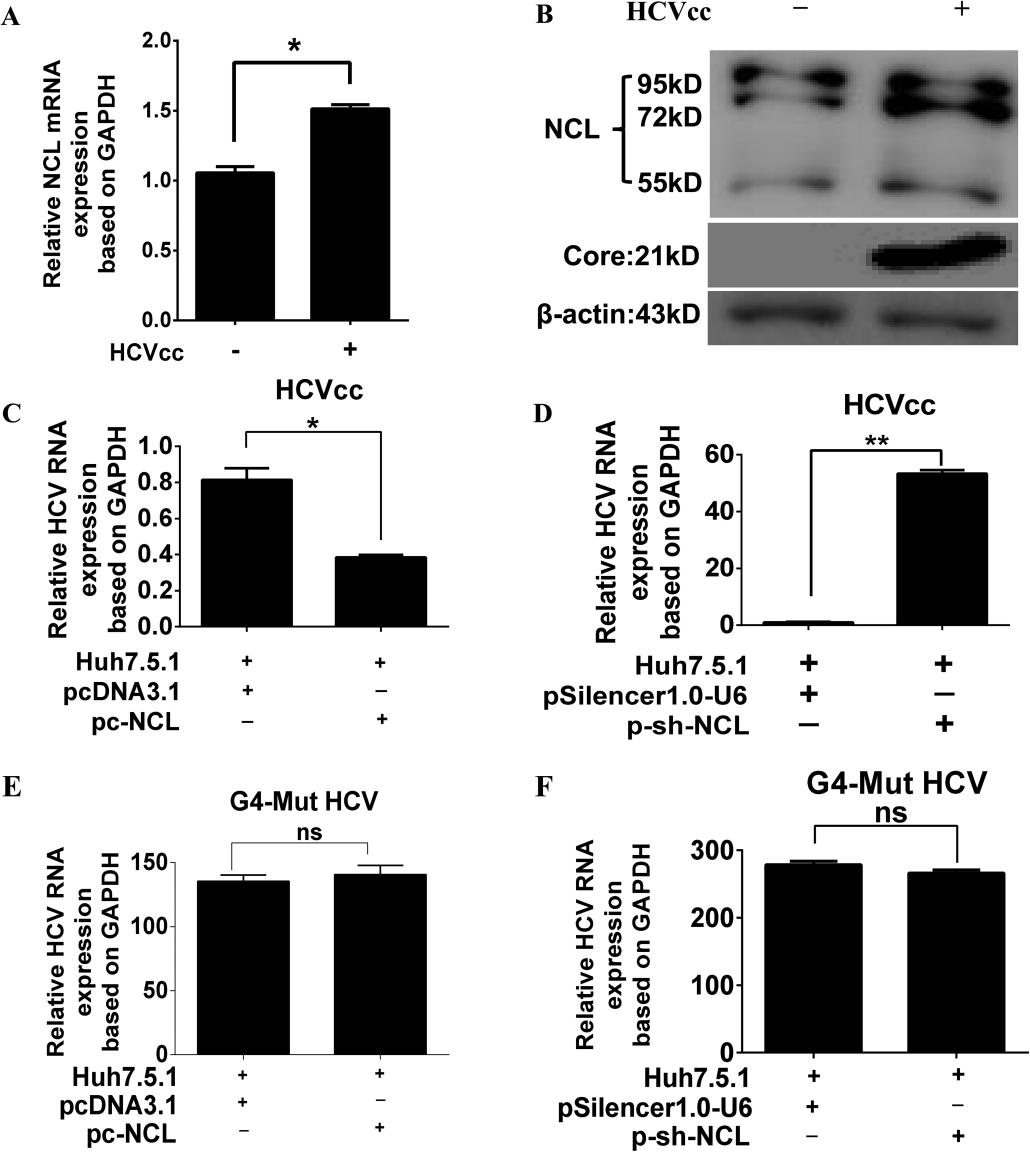
NCL suppresses HCV replication by RT-qPCR analysis. (A, B) HCV infection promotes NCL mRNA (**A**) and protein expression levels (**B**) in Huh7.5.1 and HCVcc-Huh7.5.1 cells. *P < 0.05. (**C**) Overexpression of NCL inhibited HCV RNA replication. (**D**) Knockdown of NCL promoted HCV RNA replication. (**E**) Overexpression of NCL did not affect HCV-G4-Mut RNA replication. (**F**) Knockdown of NCL did not affect HCV-G4-Mut RNA replication. GADPH was used as the internal control.

Next, we aimed to detect any alteration in the expression of NCL after HCV infection in Huh7.5.1 cells. We found that HCV infection promoted both mRNA and protein expression levels of NCL in Huh7.5.1 cells after HCV infection (Figure 7A, B).

Next, Huh7.5.1 cells were infected with JFH1 and transfected with indicated plasmids (Figure 7C–F). Overexpression of NCL (pc-NCL transfection group) dramatically decreased HCV total RNA levels in JFH1 HCV-Huh7.5.1 cells compared to the empty vector transfection group (pcDNA3.1 group) (Figure 7C), while knockdown of NCL (p-sh-NCL group) greatly increased HCV RNA expression compared with the empty vector group (pSilencer1.0-U6) (Figures 7D). More importantly, we separately delivered the transcribed J6/JFH1 and J6/JFH1-G4 Mut RNA into Huh-7.5.1 cells through electroporation. The introduced mutation in HCV core RNA G4 does not affect core protein translation, but disrupts the G4 motif and prevents G4 formation (see Materials and Methods for G4-mut sequence). The overexpression and silencing of NCL in J6/JFH1-G4-Mut RNA-Huh7.5.1 cells had no effect on the replication of G4-modified HCV by RT-qPCR (Figure 7E, F). Our results clearly showed that NCL could bind to and stabilize the viral core RNA G4, and thus exert an important inhibitory effect on HCV replication in cells. In addition, we observed that HCV-G4-Mut RNA levels were higher than those of WT HCV in Huh7.5 1 cells (Figure 7C, E). We postulate that WT HCV induced endogenous NCL which suppressed HCV replication, and endogenous NCL’s suppression of HCV replication might lead to lower levels of WT HCV RNA levels than HCV-G4-Mut.

## DISCUSSION

NCL is a eukaryotic nucleolar phosphoprotein, mainly localized in the nucleolus, but also found in the nucleoplasm, cytoplasm and cell membrane, and is involved in a variety of cellular processes. It has a unique tripartite structure, and each domain performs a specific function by interacting with DNA, RNA or protein (44); thus, NCL possesses multiple molecular functions, including intrinsic self-cleavage, DNA helicase, RNA helicase and DNA-dependent ATPase activity (44). Meanwhile, NCL participates in various biological processes, including ribosome biogenesis, cytokinesis, nucleogenesis, cell proliferation and growth, cytoplasmic-nucleolar transport of ribosomal components, transcriptional repression, replication, and signal transduction (44).

NCL has been increasingly implicated in several pathological processes, especially in tumorigenesis and viral infection (45–48). NCL was reported to stabilize G4 structures found in the HIV-1 LTR promoter (8) and c-myc DNA G4s (15,25) involved in HIV-1 infection and gene regulation of c-myc. NCL, was very recently shown to be sequestered specifically in a conformation-dependent manner by G4 present in aborted RNA transcripts arising from the expansion of the hexanucleotide repeat (GGGGCC)n (19), and Epstein-Barr virus EBNA1 mRNA (28). Here, our direct evidence firstly showed that NCL acted as a vital host protein capable of binding to the alive viral RNA G4 structure with high affinity at physiological conditions. We found that NCL bound and stabilized viral core RNA G4 and competitively blocked ASO to unlock the G4 structure (Figure 4B), which suggested that the affinity of NCL to G4 might be higher than that of ASO to G4. We propose that binding and stabilizing the HCV core RNA G4 structure of NCL is involved in represseing HCV replication by leading to premature replication stop, as shown in our previous publication (9).

Virus infection can induce IFN-α/β, while IFN-α/β can inhibit viral expression by inducing the expression of multiple anti-viral proteins. Similar to type I IFN, we found that HCV infection induced NCL expression at transcription level, but the mechanism underlying this induced upregulation need further investigation. The HCV-induced NCL suppressed HCV replication, suggesting that a potential negative feedback loop exists among HCV-NCL-HCV core G4. Our data suggest the regulation of G4 structure formation by specific protein NCL that binds G4 and hinders viral RNA replication.

The HCV core protein is considered to be a potential oncoprotein in HCV-related hepatocellular carcinoma (HCV-HCC) (49). Core protein is a highly basic, RNA-binding protein that presumably forms the viral nucleocapsid (50,51). It consists of three distinct predicted domains: an N-terminal two-thirds hydrophilic domain of approximately 120 amino acids (aa) (named domain D1) and a C-terminal one-third hydrophobic domain of 50 aa (named domain D2). The D1 domain includes numerous positively charged amino acids and is mainly involved in RNA binding (50). We found that NCL was associated with the HCV particles and might be involved in the viral capsid of the HCV life cycle. The core protein D1 domain was reported to interact with the HCV RNA genome to form viral nucleocapsid (50,51). We propose that core RNA G4, NCL, and core protein/nucleocapsid might form a 3-component complex, since we demonstrated G4 RNA bound to NCL and NCL was associated with viral particle. Further exploration of the possible roles of the 3-component complex among NCL-core RNA G4-core protein may be an interesting topic for future study.

A study by Murakami *et al.* revealed a direct interaction between NCL and HCV NS5B protein (52,53). Both the present study and that of Murakami *et al.* confirmed the interaction between HCV and NCL from different perspectives. Both interactions inhibit HCV replication.

Structural studies on G4–protein binding have been attempted by different groups. Computational studies showed that a G4 could fit into a protein cavity of HIV-integrase (54) and RecA protein (55). In an NMR-guided simulation, a nucleophosmin domain was docked into a G4 groove (56). Here, we used NMR to demonstrate that NCL, especially the RRM_3–4_-GAR domains (Figure 4C, Supplementary Figure S2), was the critical domain which binds and stabilizes the HCV core RNA G4. Structure simulation analysis (data not shown) showed that the critical residues involved in the binding between core-G4 and NCL were Gly475, Arg554, and Arg561. Other reports also showed that the C-terminus Arg554 and Arg561 of NCL bind to the G4 of c-myc (15). Based on our report and those of others, we propose that Arg554 and Arg561 might be the critical residues of NCL involved in the interaction between NCL and core RNA G4.

In summary, we demonstrate that NCL, a natural molecule of the host, plays an important role in limiting HCV replication by recognizing core RNA G4, thus representing a novel mechanism of host anti-viral immunity involving G4 structure.

## Supplementary Material

Supplementary Data
