# Supplementary material for: Binding of cellular nucleolin with the viral core RNA G-quadruplex structure suppresses HCV replication
Source: Nucleic Acids Res. 2018 Nov 20;47(1):56–68. doi: 10.1093/nar/gky1177 (PMC6326805; doi:10.1093/nar/gky1177)
Supplement: Supplementary Data [file gky1177_supplemental_files.zip › NAR_Revision_Supplementary_Information_.docx]

**Supplementary Information**

Fig. S1. SDS-PAGE and Coomassie blue staining for BG4 antibody. BG4 protein was expressed and purified from *E. coli* BL21 with plasmid pSANG10-3F-scFv-BG4 and subjected for SDS-PAGE. The results showed that BG4 was eluted best by 60 mM (red frame, for used in the experiment) and 80 mM imidazole. Unbound supernatants (residual) were used as a control.

Fig. S2. A schematic diagram of the structure of NCL protein and different truncated constructs. The full-length NCL, NCL-ΔR1 (RRM1 deleted) and NCL-R3-4C (N-terminal and RRM1-2 deleted) are presented as a rectangle, and motifs are indicated: RRM stands for RNA recognition motif; GAR stands for glycine/arginine-rich domain.

Fig. S3. Identification of the purified GST-NCL-R_3-4_C protein. (A) NCL mAb was used to detect the purified GST-NCL-R_3-4_C protein by western blot analysis. *E. coli* BL21 was used as a control. (B) GST-NCL-R_3-4_C protein was expressed and purified from *E. coli* BL21 with plasmid pGEX-KG-NCL-R_3-4_C and subjected for SDS-PAGE.

Table S1. Oligonucleotide sequences for each conserved PQS in HCV H77 genome used for CD/Tm analysis.

| **PQS**  **Name** | **Gene**  **Location** | **PQS**  **Location** | **PQS RNA Sequence**  **(5’-3’)** |
| --- | --- | --- | --- |
| Core | 253-825 | 519-540 | [GGG]cugc[GGG]u[GGG]c[GGG]au[GG] |
| E1 | 826-1401 | 1355-1372 | [GGG]cgaa[GG]uccu[GG]u[GG] |
| NS3 | 3331-5223 | 3414-3427 | [GG]u[GG]a[GGG]uga[GG] |
| NS4B | 5386-6168 | 5688-5701 | [GGGGGGG]u[GGG]u[GG] |
| NS5A | 6169-7512 | 7032-7042 | [GG]c[GG]a[GG]a[GG] |
| NS5B | 7513-9285 | 7719-7735 | [GG]a[GG]uuaa[GG]cagc[GG] |

Table S2. LC-MS/MS analysis.

| **No.** | **Protein Name** | **Protein ID (UniProt)** | **Numbers of unique peptides** | **Species** | **Protein Score^#^** |
| --- | --- | --- | --- | --- | --- |
| **1** | Nucleolin | sp\|P19338\|NUCL_HUMAN | 91 | HUMAN | 90.01 |
| **2** | Endoplasmin | sp\|P14625\|ENPL_HUMAN | 11 | HUMAN | 11.63 |
| **3** | ATP synthase subunit alpha, mitochondrial | sp\|P25705\|ATPA_HUMAN | 5 | HUMAN | 4.57 |
| **4** | Splicing factor, proline- and glutamine-rich | sp\|P23246\|SFPQ_HUMAN | 5 | HUMAN | 4.18 |
| **5** | Heat shock protein HSP 90-alpha | sp\|P07900\|HS90A_HUMAN | 3 | HUMAN | 4.17 |
| **6** | U1 small nuclear ribonucleoprotein 70 kDa | sp\|P08621\|RU17_HUMAN | 4 | HUMAN | 3.36 |

**^#^** A higher score indicates a more confident match.

Table S3. Sequences of oligonucleotides used in this study.

| **Assay** | **Name** | **Sequence (5’-3’)** |
| --- | --- | --- |
| **FRET** | HCV core RNA G4-dual | FAM-GGGCUGCGGGUGGGCGGGA-TAMRA |
|  | AS-HCV core RNA G4 | ACCCGCAGCCCUCCCGCCCACC |
| **Pull down assay** | Biotin-HCV core RNA G4 | biotin-GGGCUGCGGGUGGGCGGGA |
|  | Biotin-HCV core RNA G4-scramble control | biotin-GGACUGCGUGUGAGCGGGA |
| **Confocal**  **microscope** | HCV core RNA G4-FAM | FAM-AGGGCUGCGGGUGGGCGGGA |
|  | HCV core RNA G4-scramble-FAM | FAM-AGAGCUGCGAGUGAGCGAGA |
|  | ASO-HCV core RNA G4 | UCCCGCCCACCCGCAGCCC |
|  | probe 2a-FAM | GAGGGGCGAGAGCCUCGGGGGGACA |
|  | ASO-RNA 2a | UCCUGCCCAGCCGAGUCCC |

FAM: 6-carboxyfluorescein, TAMRA: 6-carboxy-tetramethylrhodamine
