# Supplementary figures and images for: Binding of cellular nucleolin with the viral core RNA G-quadruplex structure suppresses HCV replication
Source: Nucleic Acids Res. 2018 Nov 20;47(1):56–68. doi: 10.1093/nar/gky1177 (PMC6326805; doi:10.1093/nar/gky1177)

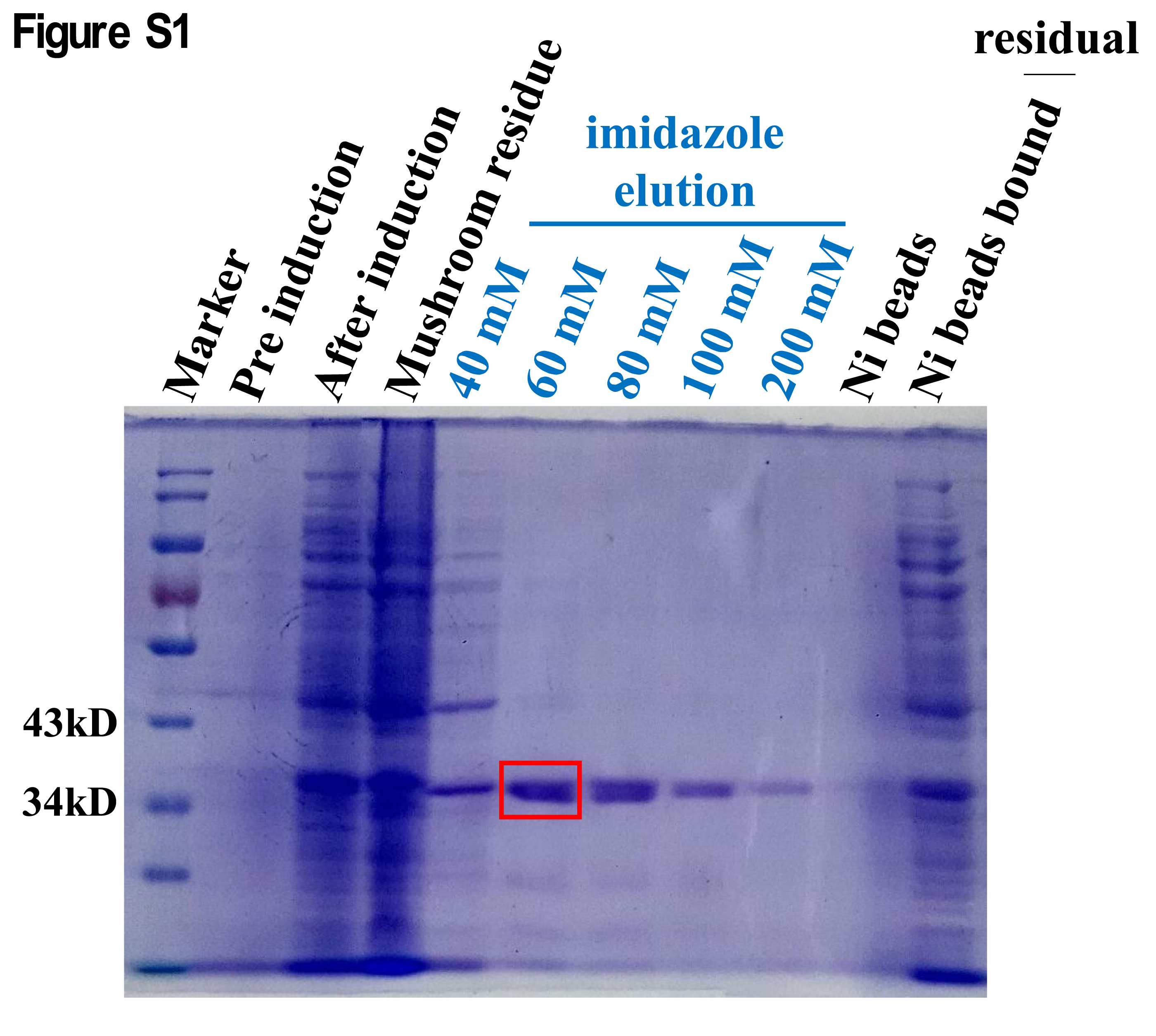

Supplement: Supplementary Data [file gky1177_supplemental_files.zip › Figure S1 NAR.tif]

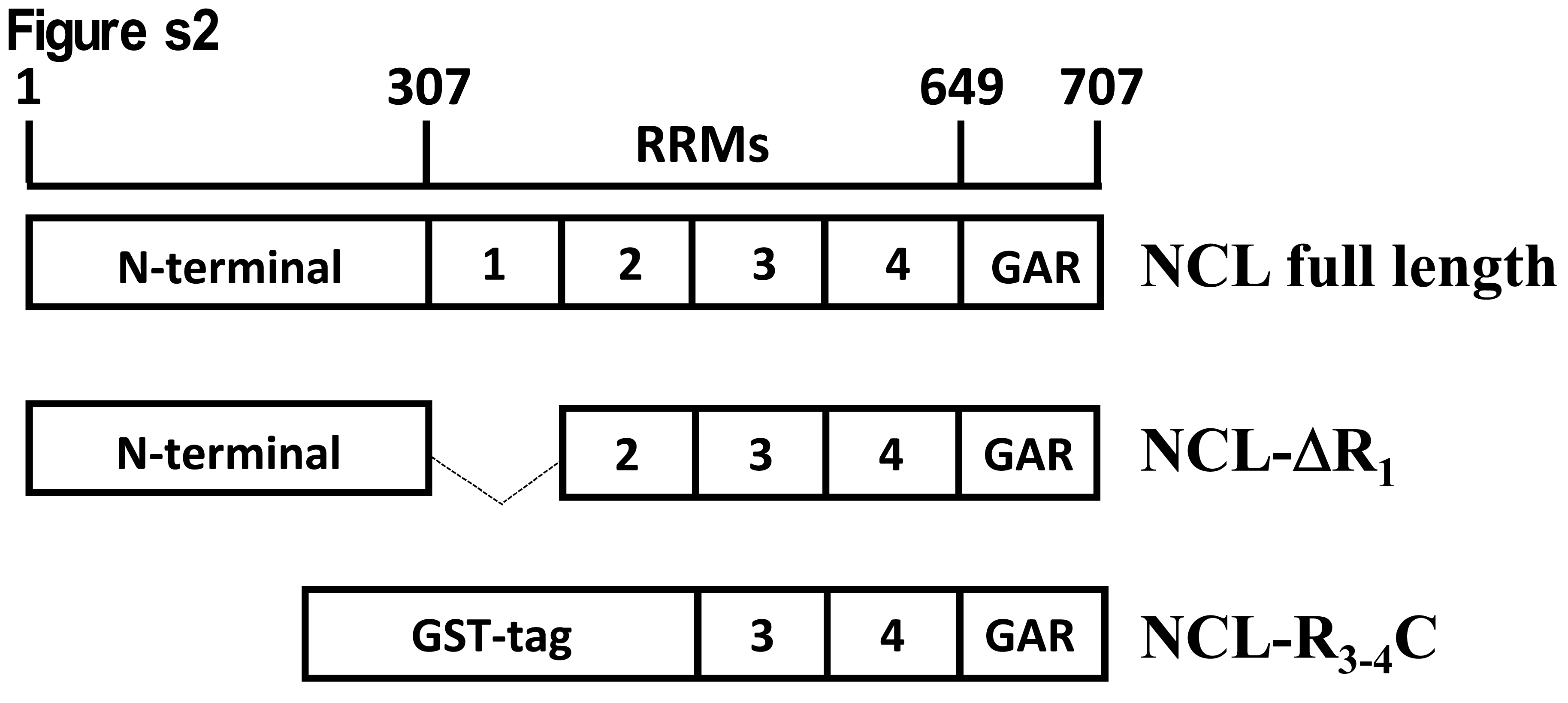

Supplement: Supplementary Data [file gky1177_supplemental_files.zip › Figure S2 NAR.tif]

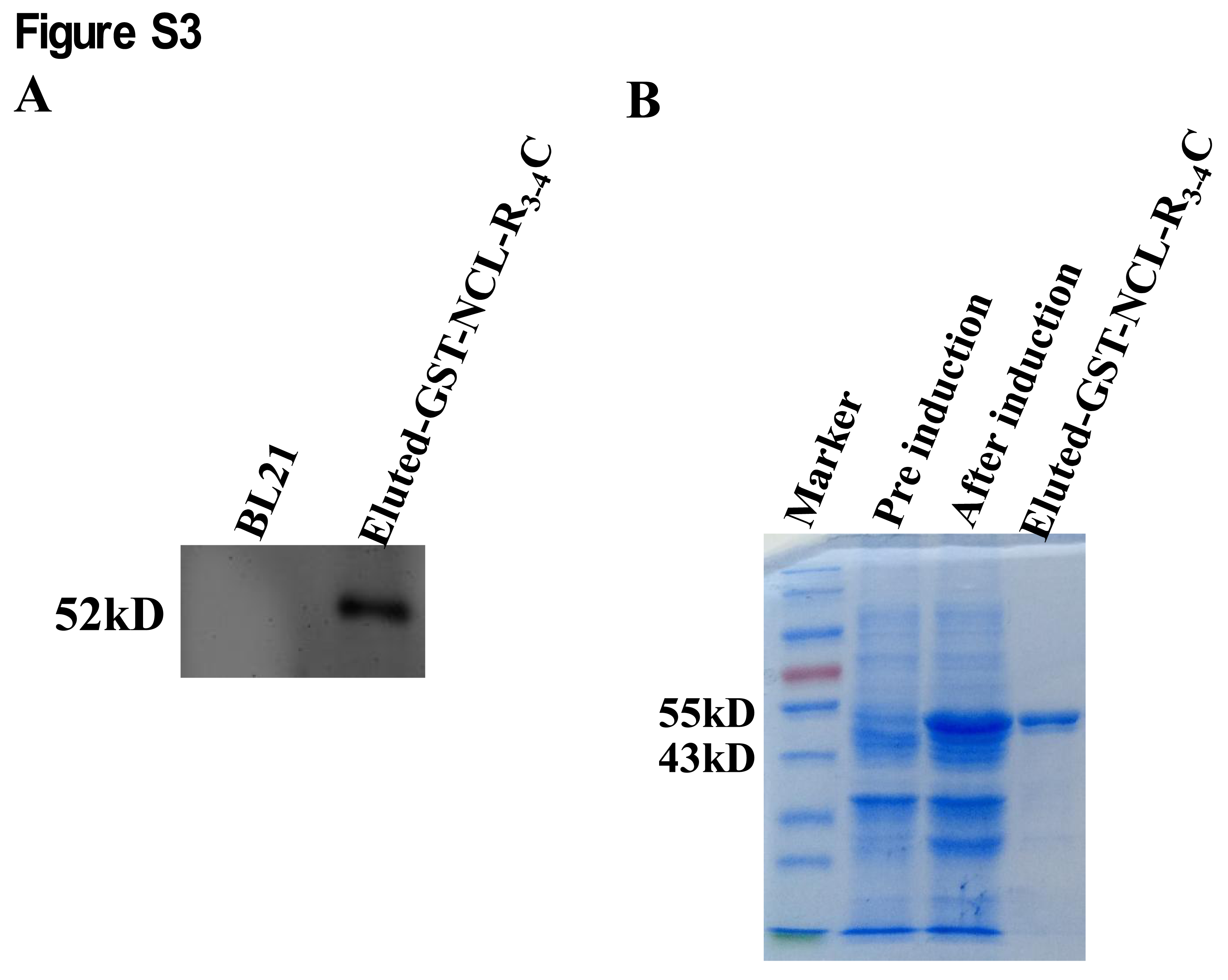

Supplement: Supplementary Data [file gky1177_supplemental_files.zip › Figure S3 NAR.tif]
